# 4-[3,5-Bis(2-hy­droxy­phen­yl)-1*H*-1,2,4-triazol-1-yl]benzoic acid dimethyl­formamide monosolvate

**DOI:** 10.1107/S1600536812005806

**Published:** 2012-02-24

**Authors:** Hoong-Kun Fun, Suchada Chantrapromma, A. S. Dayananda, H. S. Yathirajan, Saji Thomas

**Affiliations:** a X-ray Crystallography Unit, School of Physics, Universiti Sains Malaysia, 11800 USM, Penang, Malaysia; b Crystal Materials Research Unit, Department of Chemistry, Faculty of Science, Prince of Songkla University, Hat-Yai, Songkhla 90112, Thailand; c Department of Studies in Chemistry, University of Mysore, Manasagangotri, Mysore 570 006, India; d Jubilant Life Sciences Ltd, R&D Centre, C-26, Sector 59, Noida 201 301, India

## Abstract

In the mol­ecule of deferasirox dimethyl­formamide solvate, C_21_H_15_N_3_O_4_·C_3_H_7_NO, the central 1,2,4-triazole ring is tilted with respect to the benzoic acid and one of the 2-hy­droxy­phenyl units but coplanar with the other 2-hy­droxy­phenyl group, as indicated by the dihedral angles of 33.69 (9), 72.57 (8) and 5.18 (9)°, respectively. Intra­molecular O—H⋯N hydrogen bonds generate an *S*(6) ring motif. In the crystal, deferasirox mol­ecules are linked by O—H⋯N hydrogen bonds and weak C—H⋯O inter­actions into chains along the *c* axis. The dimethyl­formamide solvent mol­ecules are located between the deferasirox chains and are linked to the deferasirox mol­ecules by O—H⋯O hydrogen bonds and weak C—H⋯O inter­actions.

## Related literature

For bond-length data, see: Allen *et al.* (1987 ▶). For graph-set notation, see Bernstein *et al.* (1995 ▶). For background to, and applications of, deferasirox, see: Choudhry & Naithani (2007 ▶); Lalitha Manasa *et al.* (2011 ▶); Nick *et al.* (2003 ▶); Yang *et al.* (2007 ▶). For related structures, see: Ishak *et al.* (2011 ▶); Rajnikant *et al.* (2006 ▶); Yathirajan *et al.* (2006 ▶, 2007 ▶). For the stability of the temperature controller, see Cosier & Glazer (1986 ▶).
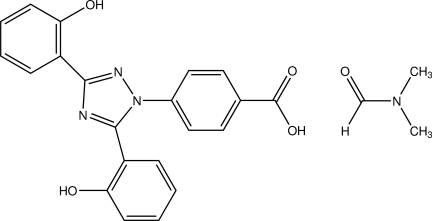


## Experimental

### 

#### Crystal data

C_21_H_15_N_3_O_4_·C_3_H_7_NO
*M* *_r_* = 446.46
Monoclinic, 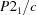

*a* = 8.8172 (8) Å
*b* = 32.669 (3) Å
*c* = 7.6900 (7) Å
β = 94.901 (2)°
*V* = 2207.0 (3) Å^3^
*Z* = 4
Mo *K*α radiation
μ = 0.10 mm^−1^
*T* = 100 K
0.32 × 0.25 × 0.11 mm

#### Data collection

Bruker APEX DUO CCD area-detector diffractometer
Absorption correction: multi-scan (*SADABS*; Bruker, 2009 ▶) *T*_min_ = 0.970, *T*_max_ = 0.989
18347 measured reflections
6379 independent reflections
4384 reflections with *I* > 2σ(*I*)
*R*_int_ = 0.038

#### Refinement

*R*[*F* ^2^ > 2σ(*F* ^2^)] = 0.058
*wR*(*F* ^2^) = 0.144
*S* = 1.04
6379 reflections
303 parameters
H atoms treated by a mixture of independent and constrained refinement
Δρ_max_ = 0.59 e Å^−3^
Δρ_min_ = −0.56 e Å^−3^

### 

Data collection: *APEX2* (Bruker, 2009 ▶); cell refinement: *SAINT* (Bruker, 2009 ▶); data reduction: *SAINT*; program(s) used to solve structure: *SHELXTL* (Sheldrick, 2008 ▶); program(s) used to refine structure: *SHELXTL*; molecular graphics: *SHELXTL*; software used to prepare material for publication: *SHELXTL* and *PLATON* (Spek, 2009 ▶).

## Supplementary Material

Crystal structure: contains datablock(s) global, I. DOI: 10.1107/S1600536812005806/ez2281sup1.cif

Structure factors: contains datablock(s) I. DOI: 10.1107/S1600536812005806/ez2281Isup2.hkl

Supplementary material file. DOI: 10.1107/S1600536812005806/ez2281Isup3.cml

Additional supplementary materials:  crystallographic information; 3D view; checkCIF report

## Figures and Tables

**Table 1:** Hydrogen-bond geometry (Å, °)

*D*—H⋯*A*	*D*—H	H⋯*A*	*D*⋯*A*	*D*—H⋯*A*
O2—H1O2⋯O5^i^	0.90	1.68	2.583 (3)	173
O3—H1O3⋯N2	0.91 (3)	1.83 (3)	2.645 (2)	148 (2)
O4—H1O4⋯N3^ii^	1.00	1.79	2.7548 (19)	161
C2—H2*A*⋯O2^iii^	0.95	2.51	3.340 (3)	146
C12—H12*A*⋯O1^iv^	0.95	2.59	3.436 (2)	149
C15—H15*A*⋯O4^v^	0.95	2.48	3.385 (2)	160
C22—H22*A*⋯O1^vi^	0.95	2.42	3.085 (3)	127

Symmetry codes: (i) 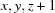; (ii) 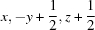; (iii) 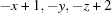; (iv) 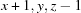; (v) 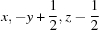; (vi) 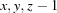.

**Fig. 1.**
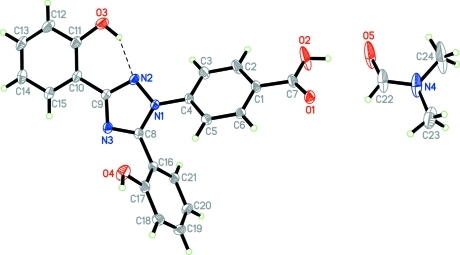
The molecular structure of the title compound, showing 50% probability displacement ellipsoids and the atom-numbering scheme. The intramolecular O—H···N hydrogen bond is shown as a dashed line.

**Fig. 2.**
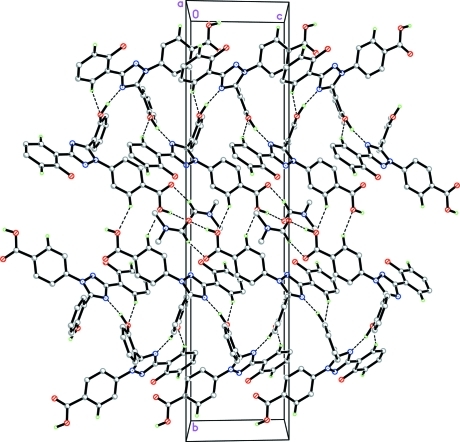
The crystal packing of the title compound viewed along the a axis. For the sake of clarity, only H atoms involved in hydrogen bonds are shown. Hydrogen bonds are drawn as dashed lines.

## supplementary crystallographic information

### Comment

The main use of deferasirox (IUPAC name: [4-[3,5-bis(2-hydroxyphenyl)-1H-1,2,4-triazol-1-yl]benzoic acid) is to reduce chronic iron overload in patients who are receiving long-term blood transfusions. Reviews on the use of deferasirox (Yang *et al.*, 2007) and its structure-activity properties (Nick *et al.*, 2003) have been published. The current status of iron overload and chelation with deferasirox has been described (Choudhry & Naithani, 2007). Novel spectrophotometric methods for the determination of deferasirox have been reported (Lalitha Manasa *et al.*, 2011). The crystal structures of 1-2-4-triazole derivatives have been reported (Ishak *et al.*, 2011; Rajnikant *et al.*, 2006; Yathirajan *et al.*, 2006; 2007). In view of the importance of deferasirox, the crystal structure of the title compound (I) is reported.

The asymmetric unit of (I), C_21_H_15_N_3_O_4_.C_3_H_7_NO, comprises the deferasirox molecule and one dimethylformamide solvent molecule. In the deferasirox molecule, the central 1,2,4-triazole ring makes the dihedral angles of 33.69 (9), 5.18 (9) and 72.57 (8)° with the C1–C6, C10–C15 and C16–C21 benzene rings respectively, indicating that the 1,2,4-triazole ring is tilted with respect to the benzoic acid (C1–C6 ring) and one of the 2-hydroxyphenyl (C10–C15 ring) moieties, whereas it is co-planar with the other 2-hydroxyphenyl (C16–C21 ring) moiety.

An intramolecular O3—H1O3···N2 hydrogen bond generates an S(6) ring motif (Bernstein *et al.*, 1995) which helps to stabilize the planarity of the 1,2,4-triazole and 2-hydroxyphenyl moiety (C8–C15/N1–N3), with the *r.m.s* = 0.342 (2) Å for the twelve non-H atoms. The dihedral angle between the two 2-hydroxyphenyl rings is 76.56 (8)°, whereas the C1–C6 ring of the benzoic acid makes the dihedral angles of 38.04 (9) and 67.34 (8)° with the C10–C15 and C16–C21 rings, respectively. Bond distances of (I) are in normal range (Allen *et al.*, 1987) and comparable with the related structures (Ishak *et al.*, (2011); Rajnikant *et al.*, (2006) and Yathirajan *et al.*, (2006; 2007).

In the crystal packing (Fig. 2), the molecules of deferasirox are linked by intermolecular O—H···N hydrogen bonds and weak C—H···O interactions (Table 1) into chains along the *c* axis. The dimethylformamide solvent molecules are located at the interstitials of the deferasirox chains and linked to the deferasirox molecules by O—H···O hydrogen bonds and weak C—H···O interactions (Fig. 2 and Table 1).

### Experimental

The title compound was obtained as a gift sample from Jubilant Life Sciences, Noida, India. Colorless block-shaped single crystals of the title compound suitable for X-ray structure determination were recrystalized from toluene/dimethylformamide (1:1 v/v) by slow evaporation of the solvent at room temperature after several days. Mp. 539–540 K.

### Refinement

Atom H1O3 of the hydroxy group was located from the difference map and refined isotropically. The remaining H atoms were positioned geometrically and allowed to ride on their parent atoms, with d(O-H) = 0.90 and 1.00 Å, d(C-H) = 0.95 Å for aromatic and sp^2^ CH, and 0.98 Å for CH_3_ atoms. The *U*_iso_ values were constrained to be 1.5*U*_eq_ of the carrier atom for methyl H atoms and 1.2*U*_eq_ for the remaining H atoms. A rotating group model was used for the methyl groups.

### Crystal data

**Table:**

C_21_H_15_N_3_O_4_·C_3_H_7_NO	*F*(000) = 936
*M**_r_* = 446.46	*D*_x_ = 1.344 Mg m^−^^3^
Monoclinic, *P*2_1_/*c*	Melting point = 539–540 K
Hall symbol: -P 2ybc	Mo *K*α radiation, λ = 0.71073 Å
*a* = 8.8172 (8) Å	Cell parameters from 6379 reflections
*b* = 32.669 (3) Å	θ = 2.3–30.0°
*c* = 7.6900 (7) Å	µ = 0.10 mm^−^^1^
β = 94.901 (2)°	*T* = 100 K
*V* = 2207.0 (3) Å^3^	Block, colorless
*Z* = 4	0.32 × 0.25 × 0.11 mm

### Data collection

**Table:**

Bruker APEX DUO CCD area-detector diffractometer	6379 independent reflections
Radiation source: sealed tube	4384 reflections with *I* > 2σ(*I*)
Graphite monochromator	*R*_int_ = 0.038
φ and ω scans	θ_max_ = 30.0°, θ_min_ = 2.3°
Absorption correction: multi-scan (*SADABS*; Bruker, 2009)	*h* = −12→11
*T*_min_ = 0.970, *T*_max_ = 0.989	*k* = −44→45
18347 measured reflections	*l* = −10→10

### Refinement

**Table:**

Refinement on *F*^2^	Primary atom site location: structure-invariant direct methods
Least-squares matrix: full	Secondary atom site location: difference Fourier map
*R*[*F*^2^ > 2σ(*F*^2^)] = 0.058	Hydrogen site location: inferred from neighbouring sites
*wR*(*F*^2^) = 0.144	H atoms treated by a mixture of independent and constrained refinement
*S* = 1.04	*w* = 1/[σ^2^(*F*_o_^2^) + (0.0461*P*)^2^ + 1.5844*P*] where *P* = (*F*_o_^2^ + 2*F*_c_^2^)/3
6379 reflections	(Δ/σ)_max_ = 0.001
303 parameters	Δρ_max_ = 0.59 e Å^−^^3^
0 restraints	Δρ_min_ = −0.56 e Å^−^^3^

### Special details

**Table:**

Experimental. The crystal was placed in the cold stream of an Oxford Cryosystems Cobra open-flow nitrogen cryostat (Cosier & Glazer, 1986) operating at 100.0 (1) K.
Geometry. All esds (except the esd in the dihedral angle between two l.s. planes) are estimated using the full covariance matrix. The cell esds are taken into account individually in the estimation of esds in distances, angles and torsion angles; correlations between esds in cell parameters are only used when they are defined by crystal symmetry. An approximate (isotropic) treatment of cell esds is used for estimating esds involving l.s. planes.
Refinement. Refinement of F^2^ against ALL reflections. The weighted R-factor wR and goodness of fit S are based on F^2^, conventional R-factors R are based on F, with F set to zero for negative F^2^. The threshold expression of F^2^ > 2sigma(F^2^) is used only for calculating R-factors(gt) etc. and is not relevant to the choice of reflections for refinement. R-factors based on F^2^ are statistically about twice as large as those based on F, and R- factors based on ALL data will be even larger.

### Fractional atomic coordinates and isotropic or equivalent isotropic displacement parameters (Å^2^)

**Table:**

	*x*	*y*	*z*	*U*_iso_*/*U*_eq_	
O1	0.33110 (15)	0.08811 (4)	1.31456 (16)	0.0238 (3)	
O2	0.3878 (3)	0.02777 (5)	1.2036 (2)	0.0742 (8)	
H1O2	0.3513	0.0199	1.3047	0.111*	
O3	0.90883 (15)	0.10202 (5)	0.38917 (18)	0.0311 (3)	
H1O3	0.830 (3)	0.1058 (8)	0.456 (3)	0.047*	
O4	0.46909 (15)	0.25263 (4)	0.60966 (19)	0.0306 (3)	
H1O4	0.4693	0.2783	0.6808	0.046*	
O5	0.2916 (3)	−0.00057 (5)	0.4879 (2)	0.0792 (8)	
N1	0.52636 (15)	0.14408 (4)	0.56919 (18)	0.0167 (3)	
N2	0.64907 (15)	0.13126 (5)	0.48553 (18)	0.0198 (3)	
N3	0.52841 (16)	0.18321 (4)	0.33783 (18)	0.0184 (3)	
N4	0.1441 (3)	0.01691 (6)	0.7044 (2)	0.0470 (5)	
C1	0.4152 (2)	0.08640 (5)	1.0291 (2)	0.0226 (4)	
C2	0.4791 (3)	0.06402 (6)	0.8993 (3)	0.0371 (5)	
H2A	0.4978	0.0356	0.9155	0.045*	
C3	0.5156 (2)	0.08293 (6)	0.7465 (2)	0.0302 (4)	
H3A	0.5586	0.0676	0.6579	0.036*	
C4	0.48836 (18)	0.12443 (5)	0.7255 (2)	0.0175 (3)	
C5	0.42428 (19)	0.14715 (5)	0.8531 (2)	0.0188 (3)	
H5A	0.4061	0.1756	0.8371	0.023*	
C6	0.38716 (18)	0.12784 (5)	1.0040 (2)	0.0176 (3)	
H6A	0.3420	0.1431	1.0912	0.021*	
C7	0.3750 (2)	0.06792 (5)	1.1966 (2)	0.0261 (4)	
C8	0.45577 (18)	0.17495 (5)	0.4780 (2)	0.0162 (3)	
C9	0.64624 (18)	0.15571 (5)	0.3471 (2)	0.0174 (3)	
C10	0.75991 (18)	0.15289 (5)	0.2187 (2)	0.0194 (3)	
C11	0.88427 (19)	0.12628 (6)	0.2452 (2)	0.0234 (4)	
C12	0.9916 (2)	0.12447 (7)	0.1219 (3)	0.0301 (4)	
H12A	1.0766	0.1066	0.1403	0.036*	
C13	0.9752 (2)	0.14846 (7)	−0.0266 (3)	0.0300 (4)	
H13A	1.0485	0.1469	−0.1099	0.036*	
C14	0.8524 (2)	0.17481 (6)	−0.0546 (2)	0.0274 (4)	
H14A	0.8409	0.1911	−0.1571	0.033*	
C15	0.7463 (2)	0.17722 (6)	0.0682 (2)	0.0228 (4)	
H15A	0.6631	0.1956	0.0499	0.027*	
C16	0.31623 (19)	0.19581 (5)	0.5244 (2)	0.0178 (3)	
C17	0.3273 (2)	0.23590 (5)	0.5890 (2)	0.0210 (3)	
C18	0.1957 (2)	0.25621 (6)	0.6290 (2)	0.0247 (4)	
H18A	0.2015	0.2836	0.6701	0.030*	
C19	0.0568 (2)	0.23642 (6)	0.6089 (2)	0.0253 (4)	
H19A	−0.0323	0.2503	0.6376	0.030*	
C20	0.0455 (2)	0.19649 (6)	0.5472 (2)	0.0240 (4)	
H20A	−0.0506	0.1832	0.5341	0.029*	
C21	0.17560 (19)	0.17613 (5)	0.5049 (2)	0.0203 (3)	
H21A	0.1687	0.1488	0.4627	0.024*	
C22	0.2029 (3)	0.02297 (7)	0.5528 (3)	0.0466 (6)	
H22A	0.1744	0.0472	0.4900	0.056*	
C23	0.0407 (3)	0.04676 (10)	0.7700 (3)	0.0563 (7)	
H23A	0.0277	0.0697	0.6882	0.085*	
H23B	0.0829	0.0568	0.8841	0.085*	
H23C	−0.0583	0.0339	0.7819	0.085*	
C24	0.1866 (6)	−0.01805 (8)	0.8146 (4)	0.0949 (15)	
H24A	0.2634	−0.0343	0.7608	0.142*	
H24B	0.0965	−0.0350	0.8277	0.142*	
H24C	0.2284	−0.0085	0.9295	0.142*	

### Atomic displacement parameters (Å^2^)

**Table:**

	*U*^11^	*U*^22^	*U*^33^	*U*^12^	*U*^13^	*U*^23^
O1	0.0325 (7)	0.0215 (6)	0.0185 (6)	0.0024 (5)	0.0092 (5)	−0.0005 (5)
O2	0.173 (2)	0.0205 (8)	0.0388 (9)	0.0265 (11)	0.0645 (12)	0.0114 (7)
O3	0.0238 (6)	0.0473 (9)	0.0231 (7)	0.0112 (6)	0.0075 (5)	0.0051 (6)
O4	0.0321 (7)	0.0239 (7)	0.0376 (8)	−0.0077 (6)	0.0132 (6)	−0.0149 (6)
O5	0.182 (2)	0.0248 (8)	0.0402 (10)	0.0076 (12)	0.0640 (13)	0.0047 (7)
N1	0.0168 (6)	0.0176 (7)	0.0166 (7)	0.0009 (5)	0.0056 (5)	0.0007 (5)
N2	0.0170 (6)	0.0253 (8)	0.0178 (7)	0.0018 (6)	0.0064 (5)	0.0001 (6)
N3	0.0219 (7)	0.0173 (7)	0.0167 (7)	−0.0017 (5)	0.0052 (5)	−0.0009 (5)
N4	0.0819 (15)	0.0402 (11)	0.0216 (9)	−0.0273 (11)	0.0198 (10)	−0.0057 (8)
C1	0.0319 (9)	0.0205 (8)	0.0167 (8)	0.0051 (7)	0.0089 (7)	0.0024 (6)
C2	0.0672 (15)	0.0212 (9)	0.0263 (10)	0.0179 (10)	0.0233 (10)	0.0075 (8)
C3	0.0477 (12)	0.0240 (9)	0.0214 (9)	0.0136 (8)	0.0182 (8)	0.0032 (7)
C4	0.0181 (7)	0.0200 (8)	0.0149 (7)	0.0009 (6)	0.0033 (6)	0.0021 (6)
C5	0.0235 (8)	0.0163 (8)	0.0168 (8)	0.0009 (6)	0.0032 (6)	−0.0006 (6)
C6	0.0198 (7)	0.0179 (8)	0.0154 (7)	0.0006 (6)	0.0037 (6)	−0.0020 (6)
C7	0.0399 (10)	0.0202 (9)	0.0197 (8)	0.0056 (8)	0.0113 (8)	0.0036 (7)
C8	0.0186 (7)	0.0147 (7)	0.0158 (7)	−0.0021 (6)	0.0037 (6)	−0.0018 (6)
C9	0.0153 (7)	0.0202 (8)	0.0170 (8)	−0.0013 (6)	0.0032 (6)	−0.0016 (6)
C10	0.0167 (7)	0.0250 (9)	0.0171 (8)	−0.0035 (6)	0.0052 (6)	−0.0031 (6)
C11	0.0191 (8)	0.0336 (10)	0.0179 (8)	−0.0009 (7)	0.0037 (6)	−0.0016 (7)
C12	0.0201 (8)	0.0467 (12)	0.0243 (9)	0.0035 (8)	0.0067 (7)	−0.0028 (8)
C13	0.0228 (9)	0.0455 (12)	0.0233 (9)	−0.0048 (8)	0.0109 (7)	−0.0042 (8)
C14	0.0284 (9)	0.0344 (10)	0.0205 (9)	−0.0069 (8)	0.0083 (7)	0.0000 (8)
C15	0.0219 (8)	0.0253 (9)	0.0219 (9)	−0.0031 (7)	0.0057 (7)	−0.0009 (7)
C16	0.0222 (8)	0.0171 (8)	0.0146 (7)	0.0029 (6)	0.0052 (6)	0.0004 (6)
C17	0.0284 (9)	0.0180 (8)	0.0177 (8)	−0.0004 (7)	0.0076 (7)	−0.0005 (6)
C18	0.0355 (10)	0.0189 (8)	0.0205 (8)	0.0063 (7)	0.0076 (7)	−0.0010 (7)
C19	0.0279 (9)	0.0282 (9)	0.0209 (8)	0.0104 (8)	0.0074 (7)	0.0011 (7)
C20	0.0217 (8)	0.0292 (10)	0.0216 (9)	0.0030 (7)	0.0053 (7)	0.0008 (7)
C21	0.0241 (8)	0.0193 (8)	0.0181 (8)	0.0009 (7)	0.0061 (7)	0.0001 (6)
C22	0.0835 (18)	0.0346 (12)	0.0241 (10)	−0.0237 (12)	0.0197 (12)	−0.0027 (9)
C23	0.0472 (14)	0.093 (2)	0.0303 (12)	−0.0088 (14)	0.0127 (11)	0.0048 (13)
C24	0.222 (5)	0.0290 (13)	0.0438 (16)	−0.011 (2)	0.069 (2)	0.0036 (11)

### Geometric parameters (Å, º)

**Table:**

O1—C7	1.211 (2)	C9—C10	1.468 (2)
O2—C7	1.317 (2)	C10—C15	1.401 (2)
O2—H1O2	0.9032	C10—C11	1.401 (2)
O3—C11	1.364 (2)	C11—C12	1.397 (2)
O3—H1O3	0.91 (3)	C12—C13	1.382 (3)
O4—C17	1.361 (2)	C12—H12A	0.9500
O4—H1O4	1.0018	C13—C14	1.386 (3)
O5—C22	1.233 (3)	C13—H13A	0.9500
N1—C8	1.350 (2)	C14—C15	1.387 (2)
N1—N2	1.3707 (18)	C14—H14A	0.9500
N1—C4	1.427 (2)	C15—H15A	0.9500
N2—C9	1.329 (2)	C16—C21	1.393 (2)
N3—C8	1.327 (2)	C16—C17	1.401 (2)
N3—C9	1.371 (2)	C17—C18	1.394 (2)
N4—C22	1.331 (3)	C18—C19	1.382 (3)
N4—C24	1.452 (4)	C18—H18A	0.9500
N4—C23	1.454 (4)	C19—C20	1.389 (3)
C1—C6	1.387 (2)	C19—H19A	0.9500
C1—C2	1.394 (2)	C20—C21	1.389 (2)
C1—C7	1.493 (2)	C20—H20A	0.9500
C2—C3	1.390 (3)	C21—H21A	0.9500
C2—H2A	0.9500	C22—H22A	0.9500
C3—C4	1.384 (2)	C23—H23A	0.9800
C3—H3A	0.9500	C23—H23B	0.9800
C4—C5	1.389 (2)	C23—H23C	0.9800
C5—C6	1.384 (2)	C24—H24A	0.9800
C5—H5A	0.9500	C24—H24B	0.9800
C6—H6A	0.9500	C24—H24C	0.9800
C8—C16	1.477 (2)		
			
C7—O2—H1O2	106.4	C13—C12—H12A	119.8
C11—O3—H1O3	107.8 (16)	C11—C12—H12A	119.8
C17—O4—H1O4	111.1	C12—C13—C14	120.40 (17)
C8—N1—N2	109.37 (13)	C12—C13—H13A	119.8
C8—N1—C4	129.99 (14)	C14—C13—H13A	119.8
N2—N1—C4	120.61 (13)	C13—C14—C15	119.53 (18)
C9—N2—N1	103.34 (13)	C13—C14—H14A	120.2
C8—N3—C9	103.93 (14)	C15—C14—H14A	120.2
C22—N4—C24	121.9 (2)	C14—C15—C10	121.02 (17)
C22—N4—C23	120.4 (2)	C14—C15—H15A	119.5
C24—N4—C23	117.6 (2)	C10—C15—H15A	119.5
C6—C1—C2	119.39 (16)	C21—C16—C17	120.27 (15)
C6—C1—C7	117.52 (15)	C21—C16—C8	120.88 (15)
C2—C1—C7	123.10 (16)	C17—C16—C8	118.85 (15)
C3—C2—C1	120.62 (17)	O4—C17—C18	123.84 (16)
C3—C2—H2A	119.7	O4—C17—C16	116.90 (15)
C1—C2—H2A	119.7	C18—C17—C16	119.26 (16)
C4—C3—C2	118.93 (17)	C19—C18—C17	119.96 (17)
C4—C3—H3A	120.5	C19—C18—H18A	120.0
C2—C3—H3A	120.5	C17—C18—H18A	120.0
C3—C4—C5	121.21 (16)	C18—C19—C20	121.00 (16)
C3—C4—N1	119.21 (15)	C18—C19—H19A	119.5
C5—C4—N1	119.58 (15)	C20—C19—H19A	119.5
C6—C5—C4	119.24 (16)	C19—C20—C21	119.54 (17)
C6—C5—H5A	120.4	C19—C20—H20A	120.2
C4—C5—H5A	120.4	C21—C20—H20A	120.2
C5—C6—C1	120.61 (15)	C20—C21—C16	119.95 (16)
C5—C6—H6A	119.7	C20—C21—H21A	120.0
C1—C6—H6A	119.7	C16—C21—H21A	120.0
O1—C7—O2	122.90 (17)	O5—C22—N4	124.8 (2)
O1—C7—C1	122.74 (16)	O5—C22—H22A	117.6
O2—C7—C1	114.34 (16)	N4—C22—H22A	117.6
N3—C8—N1	109.92 (14)	N4—C23—H23A	109.5
N3—C8—C16	124.90 (15)	N4—C23—H23B	109.5
N1—C8—C16	125.16 (14)	H23A—C23—H23B	109.5
N2—C9—N3	113.43 (14)	N4—C23—H23C	109.5
N2—C9—C10	122.24 (15)	H23A—C23—H23C	109.5
N3—C9—C10	124.33 (15)	H23B—C23—H23C	109.5
C15—C10—C11	118.88 (15)	N4—C24—H24A	109.5
C15—C10—C9	120.31 (15)	N4—C24—H24B	109.5
C11—C10—C9	120.80 (16)	H24A—C24—H24B	109.5
O3—C11—C12	117.17 (17)	N4—C24—H24C	109.5
O3—C11—C10	123.14 (15)	H24A—C24—H24C	109.5
C12—C11—C10	119.68 (17)	H24B—C24—H24C	109.5
C13—C12—C11	120.48 (18)		
			
C8—N1—N2—C9	0.46 (17)	N3—C9—C10—C15	−4.8 (3)
C4—N1—N2—C9	178.81 (14)	N2—C9—C10—C11	−5.3 (3)
C6—C1—C2—C3	0.5 (3)	N3—C9—C10—C11	174.57 (16)
C7—C1—C2—C3	−179.4 (2)	C15—C10—C11—O3	178.67 (17)
C1—C2—C3—C4	0.4 (3)	C9—C10—C11—O3	−0.7 (3)
C2—C3—C4—C5	−0.6 (3)	C15—C10—C11—C12	0.0 (3)
C2—C3—C4—N1	179.67 (19)	C9—C10—C11—C12	−179.37 (17)
C8—N1—C4—C3	144.85 (19)	O3—C11—C12—C13	−179.39 (18)
N2—N1—C4—C3	−33.1 (2)	C10—C11—C12—C13	−0.6 (3)
C8—N1—C4—C5	−34.9 (3)	C11—C12—C13—C14	0.4 (3)
N2—N1—C4—C5	147.16 (16)	C12—C13—C14—C15	0.5 (3)
C3—C4—C5—C6	0.0 (3)	C13—C14—C15—C10	−1.1 (3)
N1—C4—C5—C6	179.74 (15)	C11—C10—C15—C14	0.9 (3)
C4—C5—C6—C1	0.8 (3)	C9—C10—C15—C14	−179.77 (16)
C2—C1—C6—C5	−1.1 (3)	N3—C8—C16—C21	106.5 (2)
C7—C1—C6—C5	178.80 (17)	N1—C8—C16—C21	−71.9 (2)
C6—C1—C7—O1	−6.0 (3)	N3—C8—C16—C17	−73.7 (2)
C2—C1—C7—O1	173.9 (2)	N1—C8—C16—C17	107.98 (19)
C6—C1—C7—O2	172.4 (2)	C21—C16—C17—O4	177.93 (15)
C2—C1—C7—O2	−7.7 (3)	C8—C16—C17—O4	−1.9 (2)
C9—N3—C8—N1	0.40 (18)	C21—C16—C17—C18	−1.7 (2)
C9—N3—C8—C16	−178.18 (15)	C8—C16—C17—C18	178.41 (15)
N2—N1—C8—N3	−0.56 (18)	O4—C17—C18—C19	−178.03 (17)
C4—N1—C8—N3	−178.70 (15)	C16—C17—C18—C19	1.6 (3)
N2—N1—C8—C16	178.01 (15)	C17—C18—C19—C20	−0.7 (3)
C4—N1—C8—C16	−0.1 (3)	C18—C19—C20—C21	−0.1 (3)
N1—N2—C9—N3	−0.22 (18)	C19—C20—C21—C16	0.0 (3)
N1—N2—C9—C10	179.69 (15)	C17—C16—C21—C20	0.9 (3)
C8—N3—C9—N2	−0.10 (19)	C8—C16—C21—C20	−179.18 (16)
C8—N3—C9—C10	179.99 (15)	C24—N4—C22—O5	3.3 (4)
N2—C9—C10—C15	175.33 (16)	C23—N4—C22—O5	179.7 (3)

### Hydrogen-bond geometry (Å, º)

**Table:**

*D*—H···*A*	*D*—H	H···*A*	*D*···*A*	*D*—H···*A*
O2—H1*O*2···O5^i^	0.90	1.68	2.583 (3)	173
O3—H1*O*3···N2	0.91 (3)	1.83 (3)	2.645 (2)	148 (2)
O4—H1*O*4···N3^ii^	1.00	1.79	2.7548 (19)	161
C2—H2*A*···O2^iii^	0.95	2.51	3.340 (3)	146
C12—H12*A*···O1^iv^	0.95	2.59	3.436 (2)	149
C15—H15*A*···O4^v^	0.95	2.48	3.385 (2)	160
C22—H22*A*···O1^vi^	0.95	2.42	3.085 (3)	127

Symmetry codes: (i) *x*, *y*, *z*+1; (ii) *x*, −*y*+1/2, *z*+1/2; (iii) −*x*+1, −*y*, −*z*+2; (iv) *x*+1, *y*, *z*−1; (v) *x*, −*y*+1/2, *z*−1/2; (vi) *x*, *y*, *z*−1.

## References

[bb1] Allen, F. H., Kennard, O., Watson, D. G., Brammer, L., Orpen, A. G. & Taylor, R. (1987). *J. Chem. Soc. Perkin Trans. 2*, pp. S1–19.

[bb2] Bernstein, J., Davis, R. E., Shimoni, L. & Chang, N.-L. (1995). *Angew. Chem. Int. Ed. Engl.* **34**, 1555–1573.

[bb3] Bruker (2009). *APEX2*, *SAINT* and *SADABS* Bruker AXS Inc., Madison, Wisconsin, USA.

[bb4] Choudhry, V. P. & Naithani, R. (2007). *Indian J. Pediatr* **74**, 759–764. 10.1007/s12098-007-0134-7 17785900

[bb5] Cosier, J. & Glazer, A. M. (1986). *J. Appl. Cryst.* **19**, 105–107.

[bb6] Ishak, D. H. A., Tajuddin, H. A., Abdullah, Z., Abd Halim, S. N. & Tiekink, E. R. T. (2011). *Acta Cryst.* E**67**, o1658. 10.1107/S1600536811022409 PMC3151797 21837058

[bb7] Lalitha Manasa, P., Shanmukh Kumar, J. V., Vijaya Saradhi, S. & Rajesh, V. (2011). *Int. J. Pharm. Biomed. Res.* **2**, 1–3.

[bb8] Nick, H., Acklin, P., Lattmann, R., Buehlmayer, P., Hauffe, S., Schupp, J. & Alberti, D. (2003). *Curr. Med. Chem.* **10**, 1065–1076. 10.2174/0929867033457610 12678677

[bb9] Rajnikant, , Dinesh, , Deshmukh, M. B. & Shawney, A. (2006). *Acta Cryst.* E**62**, o1373–o1374.

[bb10] Sheldrick, G. M. (2008). *Acta* *Cryst.* A**64**, 112–122. 10.1107/S0108767307043930 18156677

[bb11] Spek, A. L. (2009). *Acta Cryst.* D**65**, 148–155. 10.1107/S090744490804362X PMC2631630 19171970

[bb12] Yang, L. P., Keam, S. J. & Keating, G. M. (2007). *Drugs*, **67**, 2211–2230. 10.2165/00003495-200767150-00007 17927285

[bb13] Yathirajan, H. S., Sarojini, B. K., Narayana, B., Sunil, K. & Bolte, M. (2007). *Acta Cryst.* E**63**, o1398–o1399.

[bb14] Yathirajan, H. S., Vijaya Raj, K. K., Narayana, B., Sarojini, B. K. & Bolte, M. (2006). *Acta Cryst.* E**62**, o4444–o4445.

